# Stent-induced new entry and device migration associated with hemodynamic stress after thoracic endovascular aortic repair for type B chronic aortic dissection using computational fluid dynamics analysis: a case report

**DOI:** 10.1186/s44215-024-00146-6

**Published:** 2024-02-22

**Authors:** Itaru Hosaka, Takeshi Uzuka, Riko Umeta, Akihiko Sasaki

**Affiliations:** Department of Cardiovascular surgery, Sunagawa City Medical Center, Nishi 4-jo Kita 3-chome 1-1, Sunagawa-shi, Hokkaido Japan

**Keywords:** Device migration, Computational fluid dynamics, Type B chronic aortic dissection, Thoracic endovascular aortic repair, Stent-graft induced new entry

## Abstract

**Background:**

Stent graft-induced new entry (SINE) after thoracic endovascular aortic repair (TEVAR) is a serious adverse event which leads to stent graft migration and rupture. SINE is known to be more frequent in cases of chronic dissection and oversizing. However, few studies have evaluated the influence of hemodynamic stress on SINE in patients with chronic dissection. Here, we report a rare case of TEVAR for chronic dissection with a dissection stent, inducing SINE 6 years after the first surgery. In addition, we analyze the hemodynamic stress for the aortic event using computational fluid dynamics (CFD) analysis.

**Case presentation:**

A 69-year-old male underwent TEVAR with left subclavian artery debranching for chronic type B aortic dissection, using a stent graft and dissection stent. The postoperative course was uneventful, but follow-up computed tomography (CT) showed that the stent graft and bare-metal stent had dislodged 4 years after surgery. The gap between the bare-metal stent and the stent graft increased over time, and the proximal edge of the bare-metal stent led to SINE at the descending aorta 6 years after surgery. We performed reintervention to cover the SINE. The patient recovered well and was discharged at 6 days postoperatively. He is currently in good condition 6 months after reintervention. CFD analysis of the patient’s CT image suggested that the local change in wall shear stress at the stent graft and dissection stent might be related to the aortic event.

**Conclusion:**

Hemodynamic stress is a factor affecting SINE and device migration. CFD may be useful for evaluating patient-specific risk of aortic events.

## Background

Device migration after endovascular aortic repair often leads to poor outcomes [1]. Especially in thoracic endovascular aortic repair (TEVAR), device migration is caused by distal stent graft-induced new entry (d-SINE) [2], which is a serious TEVAR adverse event. Early reintervention before the occurrence of d-SINE is important. However, SINE and/or device migration is difficult to predict using typical computed tomography (CT) scans.

Computational fluid dynamics (CFD) analysis can simulate flow mechanics and can be applied to the cardiovascular system when the blood is defined as a fluid. Evaluation of stent graft structure or postoperative hemodynamics after endovascular aortic repair using CFD analysis has been reported in recent studies [3, 4]. Several studies revealed that hemodynamics affect SINE and stent graft migration risk after stent graft implantation [5–7]. CFD analysis estimates hemodynamic stress using a three-dimensional (3D) model constructed from medical images [8]. However, few case reports of SINE and device migration evaluated using CFD analysis have been published, especially in cases of chronic aortic dissection [9, 10]. We report a patient-specific CFD analysis to assess the hemodynamic stress leading to SINE and stent graft migration after type B chronic aortic dissection.

## Case presentation

**Fig. 1:**
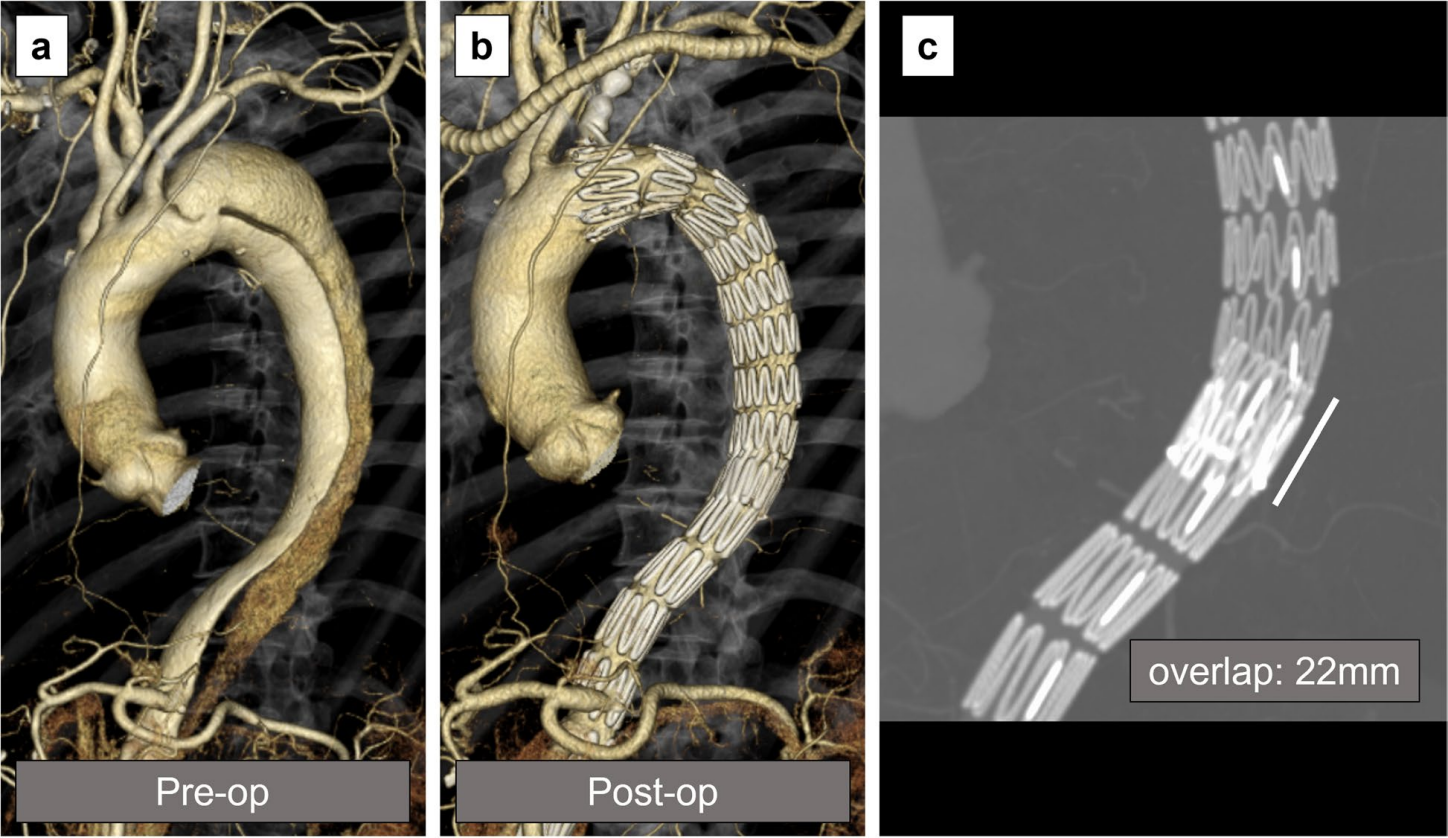
Pre- and postoperative computed tomography (CT) images of the first thoracic endovascular aortic repair (TEVAR). **a** Distal aortic arch aneurysm with type B aortic dissection; 3 years had passed since the onset. **b** Postoperative CT scan of the first TEVAR. **c** The length of the overlap with the stent graft and bare-metal stent at the first TEVAR

**Fig. 2:**
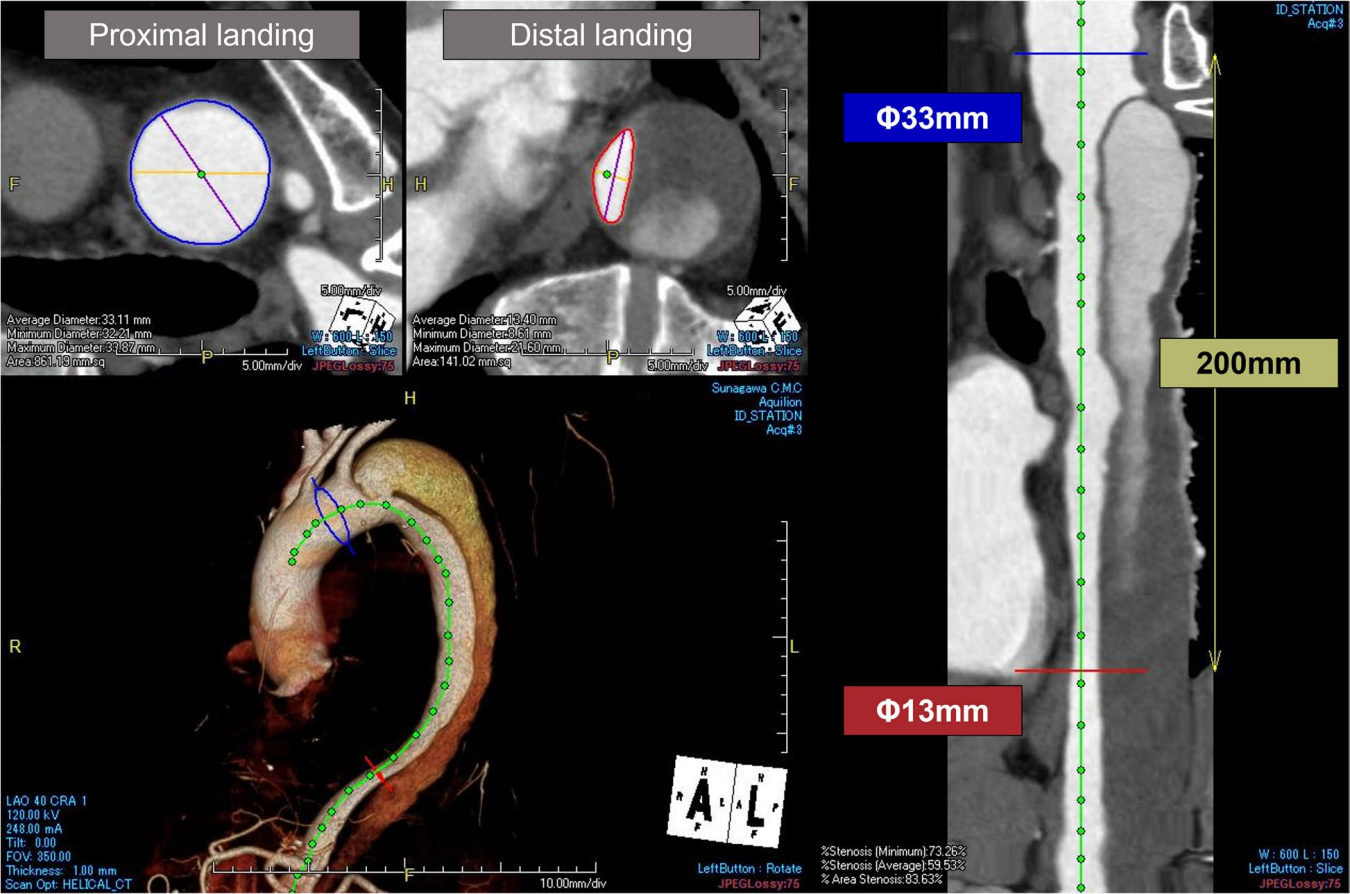
Detailed analysis of the aortic geometry. The top two panels in the left column show the average diameter of the proximal and distal landing zone. The green line in the bottom panel of the left column indicates the center line of the aorta. The right column shows the planned treatment length

**Fig. 3:**
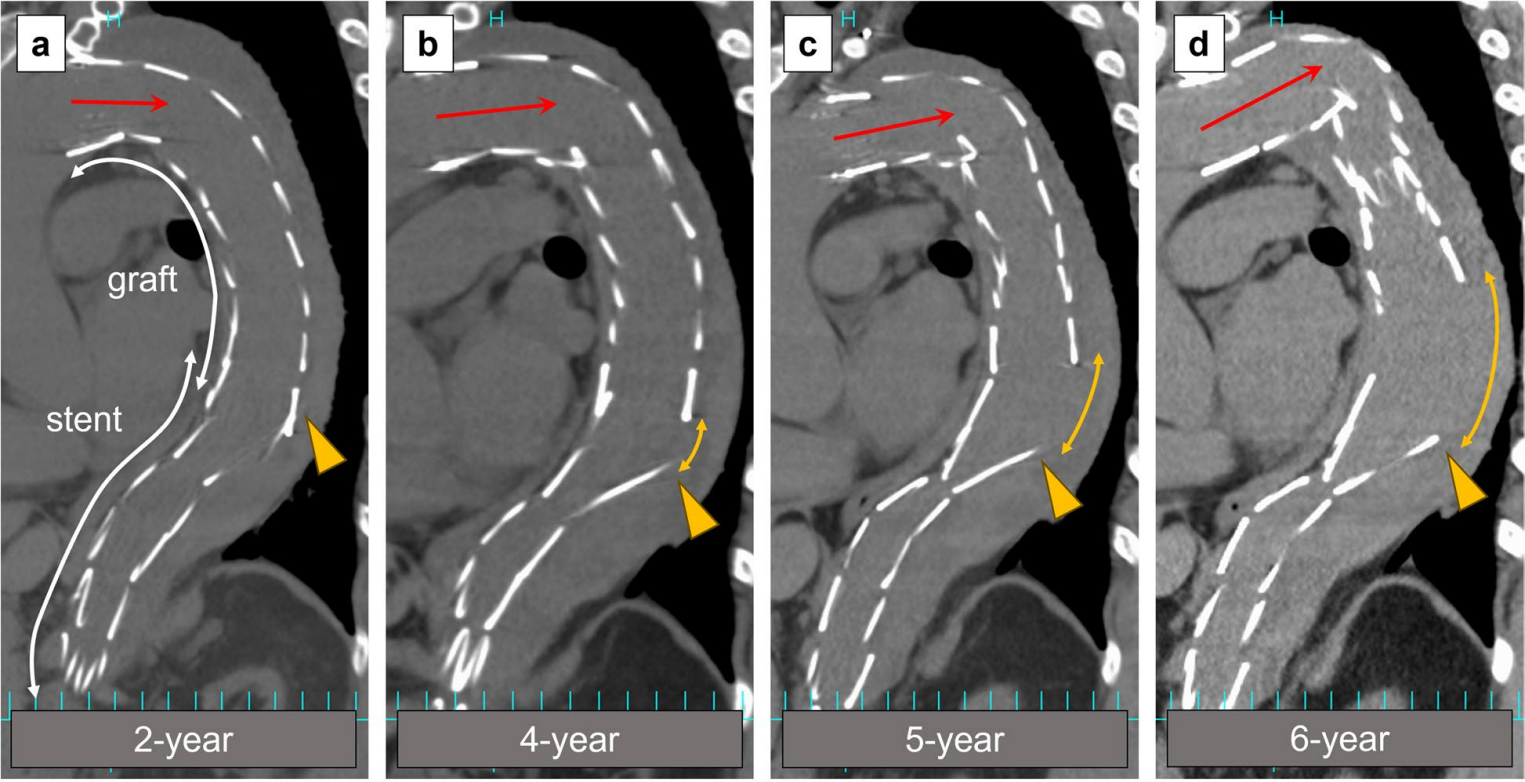
Timeline of device migration. All four panels depict the curved planar reconstruction image generated from the CT scan. These images were obtained at one postoperatively from the first TEVAR. The white line in panel (a) shows the length of the stent-graft and the bare-metal stent. The red arrow represents the direction of the proximal edge of the stent-graft, which moves cranially over time. The yellow arrowhead points to the proximal edge of the bare-metal stent, while the yellow line shows the gap between the stent-graft and the bare-metal stent. The gap was observed at 4 years after surgery and enlarged over time

**Fig. 4:**
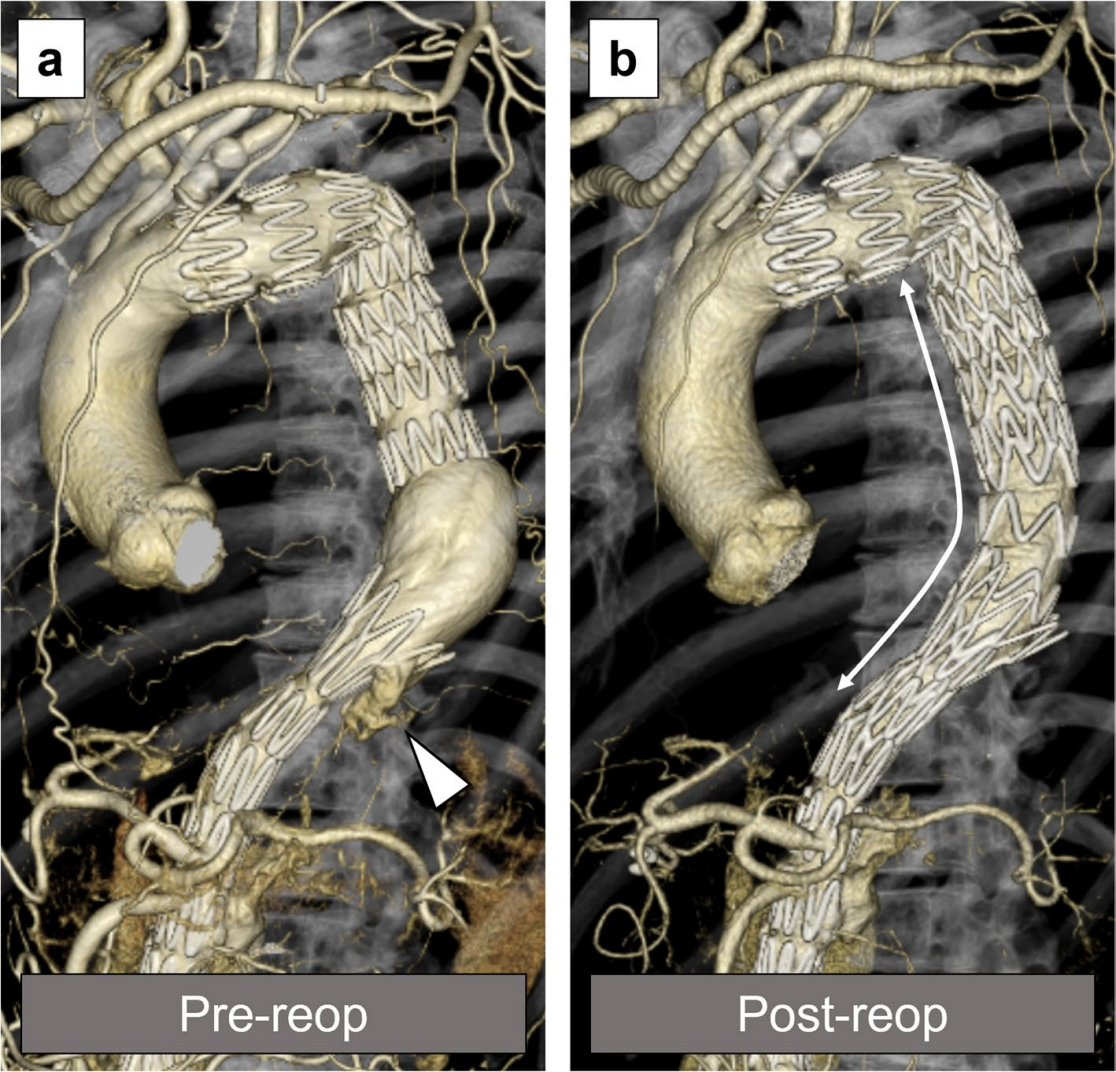
Pre- and postoperative computed tomography (CT) images of the second TEVAR. **a** Descending aortic aneurysm with stent-induced new entry (SINE) and device migration; 9 years had passed since the onset of the aortic dissection. The white arrowhead indicates the SINE. **b** postoperative CT scan after the second TEVAR showing that the SINE disappeared. The white line represents the length of the new stent-graft

A 69-year-old male developed type B aortic dissection 9 years ago. Our management of uncomplicated type B aortic dissection at that time consisted of optimal medical treatment in the acute phase. Thereafter, outpatient follow-up was performed to monitor increases in the aortic diameter, in which case the decision was made to perform surgery. The maximal aortic diameter at 6 months from the onset was 41 mm at the distal arch, increasing to 45 mm at 1 year and to 46 mm at 2 years after the onset. The patient experienced nausea and visited our emergency department 3 years after the onset. Contrast-enhanced CT scan showed true lumen narrowing and distal arch enlargement to 48 mm in the axial view (Fig. 1a). TEVAR was planned due to rapid aortic enlargement. To deploy a device at a healthy landing zone, left subclavian artery debranching was also performed (Fig. 1b). The mean proximal and distal aortic diameters were 33 mm and 13 mm, respectively, corresponding to a mean taper ratio of approximately 60% (Fig. 2). A Zenith TX2 Dissection Endovascular graft (ZTEG-2PT-36-197-PF-D) and a dissection stent (GZSD-36-164-2, both from Cook Medical, Bloomington, IN) were selected at the surgeon’s discretion. The device overlapping length was 22 mm (Fig. 1c). According to the instruction manual, Zenith dissection stent is only applied for acute type B aortic dissection. In this case, it was probably used to expand the true lumen, but further details were unknown as the medical record had been disposed of. The post-TEVAR recovery was uneventful. He was then followed as an outpatient with an annual follow-up. The patient’s postoperative course was favorable until the second year (Fig. 3a). The bare-metal stent was dislodged, and the stent graft started to move cranially in the fourth year (Fig. 3b). The gap between the bare-metal stent and stent graft increased over time (Fig. 3c). Finally, the stent graft migrated and shortened (Fig. 3d). Moreover, the proximal edge of the bare-metal stent showed a new entry into the descending aorta (Fig. 4a). Since the descending aortic aneurysm rapidly expanded to 60 mm, we performed reintervention with a Zenith alpha thoracic stent (ZTA-PT-34-30-209-W1, Cook Medical). An additional device was placed from the descending section of the previous stent graft to the third bare-metal stent to overlap the new entry (Fig. 4b). Endoleaks were not observed. The patient recovered well and was discharged at 6 days postoperatively. He is currently in good condition 6 months after reintervention.

**Fig. 5:**
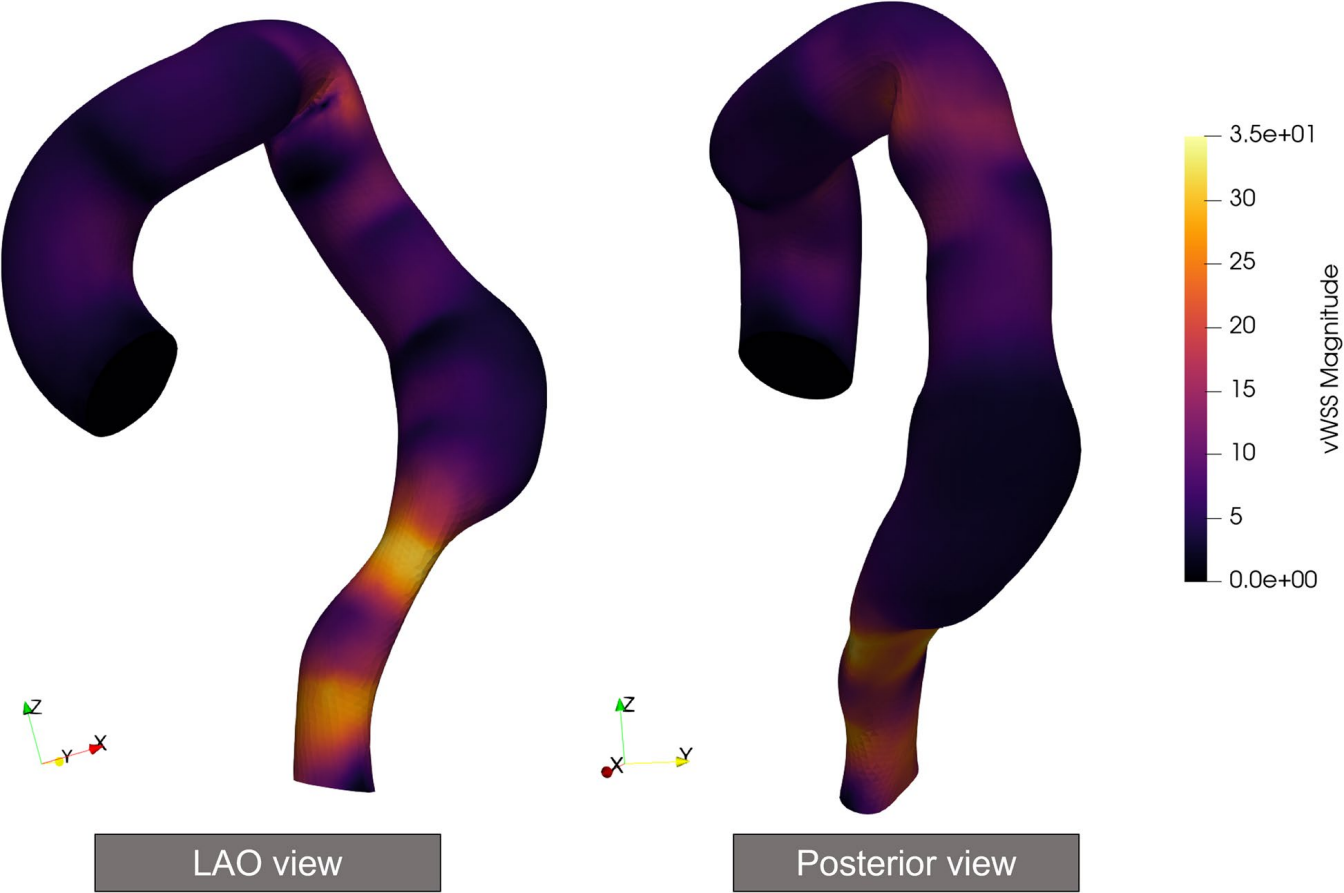
Results of the simulation. The wall shear stress (WSS) is displayed in the left anterior oblique and posterior views. A high WSS is observed at the posterior wall of the stent graft (white arrowheads) comparing with the anterior wall (black arrowheads). The WSS is increased at the second strut of the bare-metal stent consistent with the SINE location (black arrows)

We evaluated the SINE and device migration using CFD analysis performed by SimVascular [11]. Preoperative CT (Fig. 4a) was used for the analysis. First, the DICOM image was loaded into the SimVascular software. We then created a 3D model of the thoracic aorta from the DICOM image. To simplify the simulation, the brachiocephalic and left common carotid arteries were excluded from the 3D model. Blood density and viscosity were set at 1.06 g/cm/s^2^ and 0.04 g/cm^3^, respectively, which correspond to the default values for blood. The inflow rate by cardiac output was assumed as previously reported [12], and the outlet boundary condition was set to 1333 dyn·s/cm^5^ for the descending aorta as the standard value for vascular resistance. This simulation setting was referenced from the official SimVascular website (https://simvascular.github.io/documentation/flowsolver.html). The results of the simulation were visualized using ParaView (Kitware Inc., Clifton Park, NY) (Fig. 5). The median wall shear stress (WSS) at the descending aorta was 6.58 [4.56–9.82] dyn/cm^2^ and 12.70 [10.84–14.06] dyn/cm^2^ for the anterior and posterior walls of the stent graft, respectively. The median WSS at the bare-metal stent was 27.67 [24.64–29.74] dyn/cm^2^ (Fig. 5).

## Discussion

The patient’s thoracic aortic aneurysm rapidly expanded due to SINE. Risk factors for SINE are reported to be chronic dissection, high taper ratio, and high oversizing ratio [13]. Additionally, when the taper ratio is < 48% or the oversizing ratio is > 108%, SINE occurs more frequently [13]. Although an optimal oversize is needed for obtaining sufficient radial force, excessive oversize may cause an intimal tear [13]. Moreover, oversizing leads to endograft morphological changes [14], which may result in device migration [15]. In this case, the taper and oversizing ratios of the distal landing zone were 60 and 177%, respectively. Thus, this case was considered as high-risk for SINE. Finally, oversizing may have caused stent graft migration and device dislodgement over time, resulting in SINE. To prevent SINE in this case, using at least 10 mm tapered devices should have been considered to minimize the distal oversize. There should also have been a greater overlap between the stent graft and bare-metal stent at the initial TEVAR, and a dissection stent should not have been used for a chronic dissection for any reason, especially with a severe size mismatch.

In addition to endograft oversizing, WSS has been reported as a factor behind SINE occurrence [15]. The WSS was calculated using specific blood viscosity, blood velocity, and vessel radius values. WSS represents the tangential force on the arterial wall; some studies have shown that high WSS is associated with serious adverse events, including d-SINE or retrograde type A aortic dissection [15, 16]. Osswald et al. revealed that the WSS of the d-SINE group increased significantly from 8 to 15.5 dyn/cm^2^ within the stent graft [15]. Kobayashi et al. also pointed out that the mismatched shear stress between the stented and unstented intima was related to the occurrence of d-SINE [2]. Considering these reports, a high WSS at the bare-metal stent might have induced the SINE. Additionally, WSS may also be related to device migration [17]. An implanted stent graft is exposed to surface forces by in vivo hemodynamics. The surface force is called displacement force (DF), composed of a normal component by blood pressure and a tangential component by the WSS [17]. If the DF at the posterior section of the stent graft at the descending aorta is high, this force can promote cranial and dorsal migration. According to the CFD analysis, WSS within the stent graft was different between the anterior and posterior walls (Fig. 5). A high WSS at the stent graft posterior wall might be related to the migration. However, blood pressure needs to be considered for a more accurate analysis.

Various software packages can be used to analyze CFD from a 3D model. SimVascular provides a full pipeline and facilitates the calculation of blood flow velocity or WSS [18]. In addition, the simulation results can be quickly visualized using the ParaView software (https://www.paraview.org/). CFD simulations enable the assessment of hemodynamic parameters that cannot be measured using routine CT scans. Considering the results of our simulation and previous reports, focal changes in WSS could be related to aortic events after TEVAR, such as SINE and device migration. When planning device selection, it is important to determine a landing zone or treatment length that minimizes local WSS changes based on a CFD analysis. Moreover, if a device migration is once observed and WSS within a stent graft is locally high, reintervention can be performed sooner, before SINE occurs. CFD analysis may be useful for evaluating the patient-specific risk of adverse aortic events.

Nevertheless, CFD analysis has multiple limitations. First, this simulation assumed an ideal flow using results of healthy volunteers [12]. Additional analyses of pulsatile flow are needed in the future. Second, the mesh size and boundary condition influence the accuracy of the simulation but are difficult to define. In this case, the parameters were limited according to the performance of the computer.

In conclusion, we report a case of device migration due to device oversizing and SINE after TEVAR for chronic type B aortic dissection. In addition to the oversizing, we evaluated the influence of hemodynamic stress for this aortic event. The increased WSS was probably a part of the cause of device migration and SINE. CFD analysis may be useful for evaluating the patient-specific risk of aortic adverse events.

## Data Availability

Data sharing is not applicable to this article as no datasets were generated or analyzed during the current study.

## Abbreviations

3D: three-dimensional
CFD: computational fluid dynamics
CT: computed tomography
DF: displacement force
d-SINE: distal stent graft-induced new entry
TEVAR: thoracic endovascular aortic repair
WSS: wall shear stress
